# Supplementary material for: Association of multi-phase rates of force development during an isometric leg press with vertical jump performances
Source: PLoS One. 2024 Feb 21;19(2):e0292428. doi: 10.1371/journal.pone.0292428 (PMC10880963; doi:10.1371/journal.pone.0292428)
Supplement: S1 File — (PDF) [file pone.0292428.s001.pdf]

Supporting information 1. Datas and summary statistics for all participants in countermovement jumps, rebound continuous jumps, and isometric single leg press

	$IRFD_{0–50}$ ($\Delta N \cdot kg^{-1}$)	$IRFD_{50–100}$ ($\Delta N \cdot kg^{-1}$)	$IRFD_{100–150}$ ($\Delta N \cdot kg^{-1}$)	$IRFD_{150–200}$ ($\Delta N \cdot kg^{-1}$)	$IRFD20_{0–250}$ ($\Delta N \cdot kg^{-1}$)	PF ($\Delta N \cdot kg^{-1}$)	CMJ jump height (m)	RJ jump height (m)	RJ contact time (sec)	RJ-index ($m \cdot sec^{-1}$)
1	5.753	6.974	3.662	3.325	3.156	30.7	0.430	0.368	0.172	2.14
2	1.989	4.583	4.973	2.970	2.782	26.2	0.424	0.347	0.196	1.77
3	5.871	10.094	8.320	4.615	3.548	46.3	0.496	0.357	0.188	1.90
4	3.895	5.405	3.752	2.830	1.987	20.3	0.521	0.453	0.184	2.46
5	1.997	3.543	4.514	3.475	2.668	25.8	0.441	0.424	0.172	2.46
6	2.933	4.233	3.706	3.104	2.601	22.9	0.490	0.424	0.168	2.52
7	3.848	6.152	5.238	3.591	2.304	27.1	0.508	0.459	0.208	2.21
8	4.100	5.198	0.659	0.747	2.811	30.0	0.407	0.347	0.180	1.93
9	4.796	5.023	1.931	1.644	0.754	17.4	0.424	0.390	0.180	2.17
10	3.624	7.045	4.417	3.577	4.012	33.7	0.540	0.441	0.208	2.12
11	6.051	8.072	5.319	3.138	3.471	29.1	0.435	0.401	0.172	2.33
12	5.245	9.144	4.648	2.706	3.272	29.4	0.471	0.459	0.204	2.25
13	2.677	4.529	3.249	1.768	1.785	16.5	0.435	0.306	0.176	1.74
14	3.270	4.758	3.529	1.609	2.405	27.6	0.368	0.326	0.180	1.81
15	3.440	6.944	4.000	3.216	3.040	29.1	0.580	0.554	0.184	3.01
16	6.536	6.536	4.032	2.125	1.645	21.3	0.395	0.347	0.172	2.02
17	4.437	6.429	4.052	3.201	1.923	23.3	0.453	0.435	0.172	2.53
18	2.432	5.864	5.037	3.506	3.321	26.0	0.373	0.311	0.212	1.47
19	4.880	7.159	4.543	2.152	1.027	25.6	0.395	0.331	0.252	1.32
20	3.264	7.203	4.823	2.572	2.765	24.6	0.407	0.435	0.184	2.37
21	2.576	7.843	6.954	3.160	2.652	29.7	0.502	0.407	0.188	2.16
22	5.585	8.874	6.126	3.582	2.792	35.2	0.435	0.306	0.168	1.82
23	3.203	6.677	3.820	3.218	2.647	28.5	0.316	0.233	0.208	1.12
24	3.602	7.559	4.195	1.003	0.739	31.1	0.483	0.424	0.172	2.46
25	4.458	7.068	4.338	2.634	2.563	24.9	0.424	0.352	0.148	2.38
26	5.784	7.848	4.590	2.425	1.679	26.3	0.435	0.441	0.188	2.35
27	3.794	4.785	1.851	1.030	1.799	19.8	0.424	0.352	0.180	1.96
28	3.098	4.752	6.223	5.035	5.248	35.7	0.515	0.453	0.160	2.83
29	3.595	6.336	4.490	2.011	1.543	24.4	0.508	0.471	0.192	2.45
30	4.628	7.724	5.805	3.854	4.257	37.6	0.441	0.447	0.172	2.60
31	6.945	9.471	3.396	3.396	2.833	35.6	0.540	0.447	0.164	2.73
32	7.351	10.404	5.895	5.947	5.035	46.9	0.471	0.435	0.140	3.11
33	7.043	9.634	5.167	3.736	2.035	26.2	0.515	0.379	0.172	2.20
34	5.486	10.365	3.906	3.465	5.745	46.3	0.580	0.608	0.168	3.62
35	4.120	8.067	6.853	4.413	3.107	39.5	0.453	0.390	0.132	2.95
36	4.200	6.106	4.312	2.837	2.962	27.7	0.422	0.368	0.172	2.14
37	6.287	9.691	6.892	5.429	3.783	47.8	0.508	0.424	0.152	2.79
38	3.926	5.920	5.429	3.021	2.883	29.0	0.459	0.390	0.160	2.44
39	6.428	10.991	5.541	3.442	3.181	34.1	0.490	0.418	0.180	2.32
40	3.982	7.186	5.329	3.204	0.913	33.8	0.547	0.580	0.160	3.63
41	3.450	6.450	5.800	4.683	5.700	47.7	0.534	0.483	0.184	2.63
42	4.251	8.146	4.536	1.840	0.399	23.4	0.471	0.418	0.188	2.22
43	4.399	7.808	4.909	3.248	3.117	34.6	0.401	0.368	0.180	2.05
44	3.519	6.450	3.829	1.690	3.891	31.3	0.418	0.379	0.168	2.26
45	2.411	5.193	5.178	4.482	4.266	38.3	0.430	0.453	0.168	2.70
46	5.670	8.932	5.912	2.692	3.575	39.7	0.477	0.471	0.196	2.40
47	2.525	4.922	5.957	4.014	3.035	30.5	0.395	0.337	0.160	2.10
48	3.553	3.951	2.444	1.993	1.369	20.3	0.418	0.347	0.220	1.58
49	4.088	7.044	5.451	4.602	4.283	37.3	0.435	0.395	0.144	2.75
50	5.507	9.641	6.748	4.575	3.431	31.3	0.447	0.326	0.144	2.27
51	4.594	7.213	4.319	3.492	2.006	33.1	0.384	0.379	0.260	1.46
52	3.552	6.995	4.973	1.066	2.678	29.4	0.521	0.490	0.180	2.72
53	3.010	2.573	1.440	1.618	1.537	20.3	0.418	0.306	0.188	1.63
54	4.573	8.354	5.991	5.381	3.765	41.0	0.495	0.418	0.156	2.68
55	3.193	5.582	4.258	2.349	2.687	19.7	0.515	0.395	0.168	2.35
56	3.355	6.680	5.781	3.894	4.358	29.0	0.407	0.297	0.168	1.77
57	5.975	7.934	5.202	3.868	4.329	33.1	0.447	0.435	0.200	2.18
58	2.859	5.669	4.757	2.722	2.397	30.2	0.490	0.363	0.172	2.11
59	2.743	5.844	5.638	3.868	3.621	29.3	0.321	0.246	0.196	1.26
60	7.561	10.263	4.663	2.717	2.445	35.6	0.567	0.477	0.176	2.71
61	5.347	7.088	4.428	3.098	2.646	28.9	0.424	0.287	0.208	1.38
62	4.031	5.564	3.631	3.065	3.783	26.9	0.447	0.412	0.192	2.15
63	3.833	7.367	7.132	2.592	2.293	30.5	0.502	0.424	0.164	2.58

64	3.134	5.359	3.784	2.062	1.819	25.6	0.418	0.316	0.208	1.52
65	3.470	5.728	3.773	2.576	2.728	30.7	0.453	0.407	0.220	1.85
66	4.703	4.969	0.706	0.453	1.479	12.5	0.496	0.418	0.200	2.09
67	2.693	4.816	3.971	2.727	2.328	18.6	0.496	0.430	0.284	1.51
68	4.040	7.860	5.035	3.220	3.132	29.2	0.515	0.435	0.168	2.59
69	3.689	7.475	5.561	3.828	4.150	35.4	0.471	0.337	0.144	2.34
70	4.151	8.528	6.558	4.151	2.815	27.4	0.465	0.357	0.172	2.08
71	3.400	5.619	3.514	2.153	2.688	18.5	0.412	0.287	0.192	1.50
72	1.864	2.749	2.916	2.534	2.104	19.2	0.326	0.326	0.244	1.34
73	4.095	7.233	4.574	4.654	3.470	37.6	0.587	0.435	0.184	2.37
74	4.276	8.510	6.310	3.944	4.649	39.9	0.560	0.554	0.144	3.84
75	3.663	7.055	6.150	3.723	2.834	25.7	0.540	0.441	0.180	2.45
76	2.919	2.951	1.691	2.208	1.907	23.2	0.435	0.363	0.172	2.11
77	3.123	6.293	5.640	2.980	3.111	25.1	0.508	0.471	0.172	2.74
78	3.493	5.957	4.507	2.768	2.667	28.6	0.508	0.430	0.176	2.44
79	3.582	7.455	3.328	1.803	0.532	26.4	0.459	0.357	0.180	1.99
80	6.212	7.331	4.261	3.572	3.257	31.3	0.412	0.412	0.156	2.64
81	3.562	8.534	7.756	4.680	3.343	29.9	0.534	0.508	0.176	2.89
82	5.980	8.915	5.074	3.962	2.223	31.0	0.401	0.412	0.172	2.40
83	4.565	6.472	4.941	2.756	2.951	27.1	0.547	0.352	0.148	2.38
84	2.807	6.348	6.243	3.332	4.262	31.6	0.465	0.311	0.196	1.59
85	8.708	10.286	5.739	4.068	2.660	34.8	0.465	0.447	0.164	2.73
86	4.385	6.813	4.829	2.387	0.597	20.7	0.418	0.465	0.200	2.33
87	4.902	7.072	3.145	0.192	-0.413	17.8	0.547	0.496	0.216	2.30
88	3.707	8.888	6.465	2.825	3.718	33.6	0.534	0.471	0.208	2.27
89	2.896	5.757	6.122	2.778	3.167	27.2	0.465	0.390	0.204	1.91
90	3.881	5.786	4.357	3.321	2.631	33.8	0.465	0.424	0.212	2.00
91	2.300	5.086	4.788	2.637	1.824	23.2	0.418	0.250	0.220	1.14
92	3.741	4.980	3.842	2.524	1.780	20.0	0.412	0.347	0.236	1.47
93	3.394	6.345	3.491	1.924	2.205	24.5	0.502	0.395	0.196	2.02
94	2.211	2.757	1.656	0.655	0.724	15.2	0.401	0.306	0.168	1.82
95	2.446	4.409	4.328	2.500	1.505	26.0	0.407	0.326	0.164	1.99
96	2.592	6.122	6.532	3.610	3.345	32.6	0.502	0.326	0.176	1.85
97	1.680	2.782	2.926	2.381	2.303	23.8	0.418	0.306	0.188	1.63
98	3.610	6.563	2.796	0.542	1.070	18.6	0.447	0.384	0.192	2.00
99	3.237	6.586	5.279	1.654	1.736	22.4	0.483	0.352	0.204	1.73
100	3.024	5.729	5.341	2.341	1.918	21.9	0.527	0.395	0.196	2.02
101	4.793	7.098	4.489	1.696	2.283	26.7	0.459	0.502	0.204	2.46
102	3.783	6.516	4.617	2.978	3.294	28.4	0.534	0.424	0.164	2.58
103	6.697	9.800	5.512	4.369	4.492	39.9	0.567	0.540	0.160	3.38
104	4.725	7.176	5.435	3.693	2.242	24.4	0.621	0.608	0.156	3.89
105	6.150	7.023	4.511	3.218	2.812	29.4	0.547	0.502	0.176	2.85
106	4.601	7.371	5.096	3.298	4.634	38.0	0.594	0.560	0.164	3.42
107	7.704	8.407	3.630	2.626	-0.473	28.0	0.614	0.502	0.156	3.22
108	4.203	7.890	5.058	3.451	3.377	31.2	0.601	0.635	0.176	3.61
109	6.076	7.864	2.991	3.323	1.962	25.9	0.594	0.502	0.164	3.06
110	4.809	6.355	3.727	2.691	3.154	30.1	0.628	0.508	0.156	3.26
111	5.128	7.782	5.501	3.055	2.833	28.1	0.573	0.554	0.172	3.22
112	5.107	8.260	4.583	3.358	2.170	31.2	0.671	0.483	0.176	2.75
113	4.543	6.543	3.583	3.072	1.936	23.4	0.540	0.447	0.160	2.80
114	3.465	6.124	3.495	3.406	2.987	26.0	0.608	0.554	0.184	3.01
115	6.872	9.824	4.802	3.304	3.612	29.9	0.547	0.477	0.152	3.14
116	4.126	8.708	7.069	4.414	4.005	35.7	0.580	0.560	0.180	3.11
117	4.798	8.381	5.567	3.829	4.260	31.8	0.678	0.657	0.168	3.91
118	6.613	8.636	4.382	3.291	2.327	27.8	0.635	0.477	0.140	3.41
119	4.315	6.652	5.982	4.479	2.798	31.5	0.527	0.547	0.152	3.60
120	7.968	8.135	4.454	4.227	4.287	34.8	0.573	0.554	0.132	4.19
121	5.187	7.659	4.344	2.672	1.315	23.1	0.567	0.447	0.156	2.87
122	3.780	6.771	5.863	4.271	4.717	46.5	0.614	0.502	0.148	3.39
123	4.505	7.625	3.732	2.843	3.076	27.5	0.547	0.412	0.176	2.34
124	4.067	6.711	5.574	3.498	3.199	37.0	0.521	0.508	0.164	3.10
125	5.167	7.453	4.629	3.042	2.154	30.1	0.587	0.534	0.160	3.34
126	4.238	7.033	4.570	1.682	2.344	31.1	0.621	0.594	0.180	3.30
127	3.661	4.855	2.121	1.665	0.676	21.6	0.465	0.483	0.164	2.95
128	4.189	8.684	6.938	4.378	3.636	34.0	0.560	0.453	0.156	2.91
129	2.539	5.455	4.976	2.205	1.480	29.7	0.508	0.527	0.188	2.81

130	5.923	9.245	4.279	2.534	1.409	27.8	0.628	0.587	0.168	3.49
131	5.944	7.728	4.506	2.234	1.682	32.6	0.508	0.363	0.176	2.06
132	4.248	8.053	5.501	3.304	3.732	30.6	0.547	0.471	0.160	2.95
133	5.212	6.180	4.120	2.751	2.074	32.4	0.528	0.587	0.176	3.34
134	4.409	5.965	4.813	3.285	1.931	41.8	0.686	0.601	0.164	3.66
135	2.996	7.409	4.955	3.298	2.406	26.0	0.357	0.363	0.152	2.39
136	4.099	8.258	5.631	3.438	3.514	31.2	0.384	0.326	0.172	1.90
137	2.768	4.251	3.334	1.682	1.927	17.1	0.331	0.246	0.192	1.28
138	8.116	6.826	4.826	3.058	2.562	32.2	0.347	0.321	0.164	1.96
139	2.340	3.348	3.485	2.716	2.272	18.5	0.368	0.268	0.184	1.46
140	2.541	3.110	2.470	1.866	1.439	22.3	0.368	0.282	0.168	1.68
141	4.865	5.244	3.496	2.836	2.507	27.7	0.395	0.302	0.160	1.89
142	3.202	6.874	4.751	2.558	3.080	31.3	0.453	0.352	0.184	1.91
143	3.904	4.988	2.422	1.645	2.422	19.9	0.337	0.326	0.180	1.81
144	1.525	4.096	3.156	1.738	0.674	16.2	0.302	0.255	0.168	1.52
145	4.659	6.519	3.260	2.891	2.413	26.7	0.435	0.395	0.168	2.37
146	3.328	7.492	4.464	2.918	3.060	35.7	0.306	0.311	0.168	1.85
147	4.581	7.680	4.932	3.977	3.275	37.3	0.311	0.331	0.172	1.93
148	2.646	5.903	4.424	3.058	2.518	22.9	0.390	0.331	0.188	1.76
149	3.667	8.999	6.343	4.178	3.473	32.6	0.671	0.453	0.192	2.36
150	4.886	7.300	5.005	2.196	-0.188	26.9	0.527	0.407	0.224	1.82
151	4.011	7.162	5.665	4.447	2.894	36.4	0.395	0.326	0.212	1.54
152	5.388	10.974	6.782	4.242	4.686	43.6	0.671	0.521	0.180	2.90
153	4.691	8.299	4.845	1.714	1.263	28.9	0.540	0.459	0.192	2.39
154	5.085	11.565	7.109	3.639	5.184	42.5	0.573	0.390	0.172	2.27
155	4.460	6.545	3.765	3.583	4.011	43.4	0.483	0.430	0.196	2.19
156	3.698	7.024	5.295	3.775	4.365	42.5	0.534	0.471	0.132	2.66
157	3.242	6.884	4.628	3.093	2.692	24.0	0.508	0.401	0.196	2.04
158	5.176	7.664	3.908	3.237	3.771	30.1	0.407	0.401	0.168	2.39
159	5.000	10.906	5.753	3.737	3.520	37.3	0.418	0.373	0.172	2.17
160	4.137	5.877	3.949	2.905	2.169	28.8	0.459	0.418	0.164	2.55
161	2.345	4.596	3.881	1.873	1.024	21.3	0.407	0.395	0.196	2.02
162	4.734	8.118	4.659	1.851	2.656	23.7	0.363	0.363	0.172	2.11
163	4.451	6.676	3.201	2.390	2.349	24.4	0.441	0.287	0.172	1.67
164	5.246	6.691	3.960	2.659	2.384	27.4	0.395	0.384	0.172	2.24
165	3.238	5.129	2.837	2.135	0.788	20.7	0.430	0.342	0.152	2.25
166	3.257	5.599	4.711	2.275	2.450	24.1	0.441	0.326	0.176	1.85
167	3.193	7.004	2.025	-0.366	1.505	21.6	0.384	0.352	0.164	2.15
168	3.274	4.548	3.677	2.774	3.129	25.5	0.412	0.368	0.212	1.74
169	1.544	2.392	3.101	3.038	2.165	24.6	0.407	0.321	0.196	1.64
170	4.124	6.695	4.068	0.862	-0.028	20.6	0.471	0.306	0.180	1.70
171	2.140	2.974	3.672	2.671	1.973	26.9	0.447	0.255	0.156	1.63
172	2.662	4.115	3.525	2.043	1.655	19.7	0.379	0.282	0.188	1.50
173	3.562	5.736	3.896	2.375	1.823	26.0	0.401	0.342	0.160	2.14
174	4.396	6.317	2.795	0.480	2.169	30.7	0.435	0.407	0.160	2.54
175	4.628	6.858	3.913	0.982	2.370	34.3	0.508	0.430	0.192	2.24
176	2.834	5.378	5.116	3.387	3.474	33.6	0.418	0.412	0.172	2.40
177	3.436	4.495	3.322	2.231	2.134	21.9	0.379	0.326	0.176	1.85
178	6.800	8.069	3.945	4.055	2.910	34.0	0.430	0.363	0.180	2.02
179	5.627	5.941	3.102	0.858	0.264	18.1	0.490	0.379	0.172	2.20
180	4.347	5.369	2.241	0.776	1.071	13.9	0.453	0.337	0.156	2.16
181	3.688	3.582	3.564	0.230	2.074	27.2	0.465	0.459	0.172	2.67
182	4.091	6.803	6.442	4.984	3.981	35.0	0.447	0.471	0.184	2.56
183	3.052	3.748	2.548	1.526	0.622	14.1	0.459	0.326	0.176	1.85
184	5.021	8.661	4.895	2.734	2.762	35.2	0.477	0.412	0.160	2.58
185	2.587	6.641	8.714	5.507	4.387	50.2	0.580	0.435	0.156	2.79
186	3.017	5.573	4.162	3.645	3.170	30.5	0.441	0.390	0.176	2.22
187	2.820	5.362	4.992	3.713	2.589	32.0	0.412	0.220	0.188	1.17
188	5.151	7.583	4.411	1.178	0.967	24.1	0.435	0.412	0.180	2.29
189	2.089	3.211	4.504	3.486	2.415	29.7	0.490	0.435	0.200	2.18
190	4.240	3.617	2.629	2.310	2.736	25.4	0.390	0.418	0.176	2.38
191	3.382	7.326	6.404	3.209	2.834	33.4	0.483	0.465	0.164	2.84
192	2.787	4.286	2.717	1.905	1.204	17.7	0.412	0.282	0.188	1.50
193	3.348	5.378	3.526	2.119	2.207	22.5	0.352	0.321	0.188	1.71
194	3.760	5.982	3.450	1.770	1.835	20.6	0.424	0.347	0.188	1.85
195	3.348	4.473	3.832	2.735	1.952	21.0	0.496	0.384	0.176	2.18

196	3.763	3.911	2.444	0.785	1.689	20.9	0.453	0.401	0.192	2.09
197	7.060	11.030	6.716	6.537	5.403	52.2	0.459	0.521	0.148	3.52
198	3.317	8.373	6.383	3.129	3.354	36.5	0.521	0.435	0.164	2.66
199	2.828	5.043	3.986	1.755	0.170	28.2	0.347	0.331	0.184	1.80
200	5.394	7.138	4.023	2.876	2.444	25.3	0.477	0.395	0.168	2.35
201	6.609	7.754	3.648	1.259	1.745	26.8	0.459	0.471	0.132	3.57
202	2.644	5.047	4.752	2.886	1.557	30.4	0.407	0.379	0.192	1.97
203	2.709	5.581	5.718	3.571	2.709	23.1	0.441	0.441	0.188	2.35
204	3.098	7.550	6.042	4.493	4.814	39.5	0.508	0.363	0.180	2.02
205	3.881	7.822	4.296	3.037	1.778	22.0	0.471	0.412	0.156	2.64
206	4.860	7.065	5.935	3.866	5.745	43.1	0.515	0.490	0.144	3.40
207	3.092	5.157	5.029	3.861	3.889	40.0	0.459	0.430	0.164	2.62
208	2.911	4.852	4.245	3.046	2.978	22.7	0.453	0.326	0.192	1.70
209	3.501	7.703	5.238	1.919	2.157	31.3	0.490	0.477	0.180	2.65
210	3.121	5.429	4.763	3.432	3.476	35.5	0.363	0.407	0.204	1.99
211	3.845	6.729	3.773	2.023	1.779	24.3	0.465	0.363	0.216	1.68
212	4.819	5.818	2.358	1.532	1.497	25.3	0.379	0.311	0.172	1.81
213	3.657	4.722	2.870	1.590	1.281	15.6	0.412	0.412	0.152	2.71
214	3.059	4.786	4.849	3.613	3.265	31.7	0.418	0.368	0.152	2.42
215	2.500	4.770	4.013	2.253	3.882	39.3	0.352	0.287	0.152	1.89
216	4.296	2.201	2.367	1.917	1.586	18.0	0.447	0.379	0.176	2.15
217	3.752	5.088	4.605	4.392	3.596	37.1	0.435	0.379	0.156	2.43
218	4.868	6.519	3.051	3.121	2.857	24.7	0.352	0.368	0.164	2.24
219	3.800	6.788	4.388	2.325	2.063	27.0	0.540	0.447	0.188	2.38
220	5.498	5.619	2.221	-0.438	2.538	21.5	0.490	0.347	0.168	2.07
221	4.864	9.833	6.136	5.152	3.470	31.4	0.459	0.412	0.176	2.34
222	3.787	7.063	5.703	4.900	3.725	38.5	0.430	0.368	0.176	2.09
223	4.585	6.652	3.377	0.102	0.699	19.6	0.418	0.352	0.172	2.05
224	4.423	8.117	6.467	3.591	5.416	38.5	0.490	0.401	0.144	2.79
225	3.304	6.310	6.786	6.696	6.295	42.8	0.395	0.316	0.188	1.68
226	3.603	6.873	6.270	4.857	3.016	35.9	0.447	0.430	0.172	2.50
227	3.347	5.640	4.262	3.249	3.460	36.2	0.465	0.435	0.172	2.53
228	2.936	7.438	5.055	1.787	1.898	22.9	0.580	0.567	0.212	2.67
229	2.338	3.514	3.198	3.695	3.062	25.0	0.528	0.292	0.232	1.26
230	1.855	3.000	2.507	1.899	1.478	20.4	0.373	0.395	0.168	2.35
Median	3.88	6.68	4.51	2.98	2.65	28.70	0.459	0.401	0.176	2.27
QD	1.60	2.34	1.76	1.44	1.44	9.85	0.102	0.106	0.024	0.74
Min	1.52	2.20	0.66	-0.44	-0.47	12.5	0.302	0.220	0.132	1.12
Max	8.71	11.57	8.71	6.70	6.29	52.2	0.686	0.657	0.284	4.19
