# Supplementary material for: Association of multi-phase rates of force development during an isometric leg press with vertical jump performances
Source: PLoS One. 2024 Feb 21;19(2):e0292428. doi: 10.1371/journal.pone.0292428 (PMC10880963; doi:10.1371/journal.pone.0292428)
Supplement: Attachment — Submitted filename: Response to Reviewers.docx [file pone.0292428.s002.docx]

**Response to the comments**

Manuscript ID# PONE-D-21-32499

The title of manuscript:

Association of multi-phase rates of force development during an isometric leg press with vertical jump performances

----------------------------------------------------------------------------------------------------------

**Response to Reviewer # 1**

----------------------------------------------------------------------------------------------------------

**General Comments**

**The authors have completed a substantial body of work, in a very relevant athlete cohort. While the grammatical and writing style should be improved to ensure that ideas and concepts are presented as succinctly as possible, the major flaw in my opinion is the reliance placed on statistical significance of the correlation r values rather than the strength of these relationships. A stronger manuscript would be positioned around the strength or lack there of for the correlation analysis in such a large homogenous athlete cohort as compared to the very small previous cohorts that reported stronger relationships.**

[Response]

Thank you very much for taking your time to review our study. We have tried to improve grammar and writing in the manuscript as much as possible. Moreover, we have now focused on the strength of the relationship and discussed the difference compared to previous small cohort studies (L322-270, 391-395).

Specific Comment

**Ln47-48; Poor grammar**

[Response]

We have checked and revised the grammatical errors in lines 48–51.

**Ln88-94; Please reconsider the sentence length and structure as currently it is grammatically poor**

[Response]

We have checked and revised the sentence length and structure in lines 88-91.

**Ln116-118; Single sentence paragraphs are not ideal, I suggest including a statement that succinctly details what the proposed benefit to the area would be if the data is supportive of the hypothesis as method for expanding this paragraph**

[Response]

Thank you for your suggestion. We have added a note regarding the areas to which the results of this study can be applied if the hypothesis is dictated (L112-119).

**Ln121; Delete the word 'were'**

[Response]

Thank you for bringing this to our attention. We have removed the relevant text according to your instruction (L122).

**Ln122-126; Delete the words, 'of the current study'**

[Response]

Thank you for bringing this to our attention. We have removed the relevant text according to your instruction (L126).

**Ln141-142; Delete the words, 'at the long seat position'**

[Response]

Thank you for bringing this to our attention. We have removed the relevant text according to your instruction (L143-144).

**Ln142; Change the 'deg' to the symbol for degrees, change 'the' to 'their'**

[Response]

Thank you for bringing this to our attention. We have revised the relevant text according to your instruction (L140, 144-149).

**Ln142-143; Change 'being attached to' to 'against'**

[Response]

Thank you for bringing this to our attention. We have revised the relevant text according to your instruction (L144).

**Ln146-151; These three sentences have poor grammar and need to be reordered to improve the flow of information and improve the grammar and presentation**

[Response]

We have corrected the grammar and flow in the areas to which you refer. Please check the revised section (L149-153) .

**Ln154; I believe that the authors are referring to asking the participants to maintain the voluntary contraction against the pedal. Please re-word**

[Response]

Thank you for bringing this to our attention. We have corrected the sentence accordingly (L155-156).

**Ln158; Figure 1 Caption, it is unclear what the authors are referring to with the word 'allow'**

[Response]

Thank you for bringing this to our attention. The text to which you refer was a spelling error. Therefore, we have corrected it to “arrow” (L158-159, Fig 1).

**Ln 158-159; Reconsider the use of the terms 'force production' as 'contraction' seems to be a better term to be used**

[Response]

As you indicated, we changed the term to "contraction" (L156).

**Ln181; Poor grammar, please insert 'by' so the sentence reads, ".....RFD was calculated by averaging...."**

[Response]

Thank you for the suggestion. We have added "by" (L186).

**Ln190-191; I am uncertain as to why the authors have chosen to make a comparison between a single leg isometric test and a bipedal test dynamic test when the dynamic test was conducted using two force plates. Did the authors consider looking at the force time characteristics from the dominant leg recorded during the dynamic test?**

[Response]

It is noted that two force plates were used in the method in this study, which is the setup used by us to calculate performance variables and kinetics and kinematics variables for jumping exercises in our experimental facility. Although, we used to measure and process from dominate leg (L146, L195-196), trials in which leg laterality such as ground contact timing during the VJ were significantly were excluded according to the ground reaction forces of the two force plates; hence, CMJ jumping height and RJ jumping height and ground contact time were calculated correctly. Moreover, kinetics and kinematics variables and during VJs had not reported in this study.

The aim of this study was to elicudate the association between isometric RFD during explosive leg extension and dymamic force production capability during leg extension exercise, such as VJ performances, focusing on duration of force production. Therefore, RJ, which has performed within an extremely short duration (approiximately 0.1-0.2 sec), and CMJ (approiximately 0.5-1.0 sec), which has performed within a longer duration are used in the jump exercise. While IRFD is measured from single leg test, we recognize that your comment suggeste that VJ should be measured through a single leg test. However, previous studies were suggested that the contact time of a single leg test is greater than that of a double leg VJ. Further, double leg VJs are affected not only by leg extension, but also by other movements such as about hip joint abduction, which was hardly detectable in double leg VJs (Sado et al., 2020). These characteristics of the single leg VJs suggest that it is a more skilled jump than the double leg VJs, involving many factors other than plain explosive force exertion. For these reasons, we thought that a trial with bilateral jumps would be appropriate to compare with the ISLPs in this study, and the VJs in this study performed with duble leg jumps (L164-167).

**Ln192; The Bosco method is not a correct methodological reference, was this a flight-time calculation or an impulse momentum calculation?**

[Response]

The VJ variables in this study were calculated from ground contact time and flight time. Moreover, the citation was inappropriate and has been removed (L197-199).

**Ln200; I would generally prefer to see a zero before all decimal places but I understand that this may not be the journal convention, please double check the submission guidelines**

[Response]

Thank you for bringing this to our attention. We have made the addition without omitting the zero as you indicated. We have also corrected the other statistical results (L207-209).

**Ln206-214; Please indicate for the reader if any of these comparisons were statistically significant**

[Response]

Thank you for bringing this to our attention. As you indicated, we have added p-values and correlation or partial correlation coefficients for items that were significant in the results. You may review the edits in the Results section (L212-263).

**Ln220; Table 2, Please include the confidence interval in this table**

Thank you for bringing this to our attention. I have included the 95% confidence interval in Table 2 (L242-252).

**Ln233; Table 3, Again it seems strange to have correlated variables from a single leg assessment to variables (data) derived from a bipedal assessment.**

[Response]

Thank you for bringing this to our attention. Although this response contains some overlap with the previous answer, this study aimed to elicudate the relationship between IRFD during leg extension and dynamic performances with very short exercise durations, such as VJs. The single leg VJ requires more skill than a double leg VJ, as it involves many factors other than plain explosive force exertion. For this reason, double leg VJs were performed in this study. In addition, we ensured that no hip abduction or similar movements were used in the leg presses performed in this study, and trials in which these movements were observed were not used in the analysis. We have added this to the Methods (L146-148).

**Ln233; Table 3, Considering the number of participants, the relationship or lack thereof between IRFD@200-250ms and the CMJ JH is a very important finding**

Thank you for bringing this to our attention. As you indicated, we have added a discussion of the relationship between IRFD200-250 and CMJ-JH (L323-329).

**Ln248; Table 4, While some of these partial correlations are statistically significant they are small to moderate at best. Can the confidence interval be included, so the reader can be given an indication as to the spread of the relationship.**

[Response]

Thank you for your suggestion.Unfortunately, we could not find the calculation of confidence intervals from partial correlation analysis in the statistical analysis software used for the partial correlation analysis.

**Ln272; Change the word 'to' to 'with' so the sentence should read, "... which is consistent with a previous study..."**

Thank you for bringing this to our attention. We have corrected it (L288).

**Ln269-291; Unfortunately again this section is all about making comparisons between a single leg isometric metric compared to a bipedal dynamic assessment. The authors should consider conducting a single v single leg metric comparison.**

[Response]

Thank you for bringing this to our attention. As you point out,It is generally reasonable to examine the rerationship between a single leg IRFD to singl leg VJs performances. In accordance with the answer to your previous comment, the mechanics and complexities of a single leg VJ may prevent a participant from producing explosive leg extension forces; thus, the use of a double leg jump was more appropriate for achieving the purpose of this study. Although kinetic and kinematics variables were not reported in this study, there was no leg laterality to be considered. Therefore, the present study did not consider it problematic to conduct a double leg VJ and to measure IRFD with a single leg trial.

**Ln293; While the authors have hilghghited that the correlation coefficient is line but compared value is substantially higher, please provide a critical comparison and contrast point to this result**

[Response]

Thank you for bringing this to our attention. We have added a discussion of the lower correlation coefficients compared with those of previous studies (L308-329).

**Ln304; Unfortunately the authors have relied very heavily on the statistical significance of the correlation outcomes, however a major factor to these significant correlations is the large sample size, the actual r value is moderate at best and should be more strongly reflected in the level o discussion and interpretation throughout the manuscript**

[Response]

Thank you for bringing this to our attention. The text has been amended to take into account that the correlation coefficient is moderate.

**Ln309; Please insert the actual r value in brackets after IRFD to provide the reader with immediate context rather than needing to refer to the table**

[Response]

Thank you for bringing this to our attention. We have included the r values (L309, 324, 336, 338).

**Ln313-316; This sentence has poor grammar please reword,**

[Response]

We have corrected the grammar and flow in the areas you pointed out (L345-346).

Thank you for reviewing.
