# Supplementary material for: Association of multi-phase rates of force development during an isometric leg press with vertical jump performances
Source: PLoS One. 2024 Feb 21;19(2):e0292428. doi: 10.1371/journal.pone.0292428 (PMC10880963; doi:10.1371/journal.pone.0292428)
Supplement: Attachment — Submitted filename: Response to Reviwer.docx [file pone.0292428.s003.docx]

**Response to the comments**

Manuscript ID# PONE-D-21-32499

The title of manuscript:

Association of multi-phase rates of force development during an isometric leg press with vertical jump performances

**Reviewer #1: I thank the authors for the responses to the first review and the work undertaken to improve the readability of the manuscript. By positioning the interpretation on the strength of relationship as compared to the significance greatly improves the application of the outcomes. The remaining concerns are primarily related to clarifying the methodology.**

----------------------------------------------------------------------------------------------------------

**Response to Reviewer # 1**

----------------------------------------------------------------------------------------------------------

**Ln 52; "evaluated via rate of force development (RFD) of a force-time curve generated during..."**

[Response]

Yes, thank you for pointing it out. I corrected it. (L55)

**Ln 61; 'stronger'**

[Response]

Yes, thank you for pointing it out. I corrected it. (L64)

**Ln 134-156; The authors highlighted in the introduction that IMTP and ISq have been used in the past but that this may be limited by upper body grip strength of compromised due to injury concerns, however each of these tests have also had the reliability and validity of the methods comprehensively reported. As such that authors need to include what is the CV%, and ICC for repeated testing using the MST device and also justify why the 100N pre-tension has no impact. The citation provided (Ref #14) is on a very different device and procedure.**

[Response]

I stated CV% and ICC of IRFDs and PF in the statistical analysis and results (Ln 219-225, Ln 236-239, Table 3). Influence regarding pre-tension has been included in the Ln 229-234, Table 2 of the manuscript. We also reported the impact of pre-tension on IRFD using correlation coefficients and discussed the potential contribution of pre-tension to improving the ICC and CV% of IRFD (L314-328).

**Ln 165; "shingle"?**

[Response]

Yes, thank you for pointing it out. It was a simple spelling mistake. I corrected it. (L170)

**Ln 197-198; The free fall formula is an unusual formula to be applied in this context because you don't know when the object began to fall, you are only aware of the total time in the air. Application of the impulse momentum theorem, and the Law of the Conservation of Energy. Please provide a reference for the formula and explain the constant.**

[Response]

One of general method for calculating jump height in field tests is to use the total time in the air, and its reliability and validity have been demonstrated in previous research (García-López et al., 2005; Healy et al., 2016; Montalvo et al., 2021; Zushi et al., 2022). In addition, a very high correlation has been found between performance calculated from ground reaction forces during VJ and performances calculated from the total time in the air(Aragón, LF. 2000). There facts suggest that the difference in calculation methods is unlikely to have a significant effect on the results of this study. To demonstrate that we have controlled the factors that affect the calculation of jump height using the total time in the air. (L173-180)

----------------------------------------------------------------------------------------------------------

**Response to Reviewer # 2**

----------------------------------------------------------------------------------------------------------

**General comments**

**A positive aspect of the study is that the authors used a rebound jump test which has not been frequently examined in the literature and measured a large sample of subjects. In general low to moderate correlations were found between IRFDs measured at different time points and VJ performance parameters. However, the authors conclude that “the early phase IRFD measured by ISLP could enable us to assess multiple aspects of leg extension strength characteristics, which will be useful to monitor the effect of strength training on an improvement of leg extension strength capabilities”. I do not believe that the data presented support such a strong statement. The authors should make changes in their discussion considering that IRFD measurements during a single leg press explain 3 to 13% of the variance in VJ performance.**

**In addition, the manuscript is not well written and is very confusing for the reader to understand the rationale of the authors and the procedures used for testing. The manuscript should be carefully checked for English language grammar and syntax.**

[Response]

Thank you for reviewing our study. I have revised the specific comments based on the review provided in the general comments.

**Specific comments**

**Lines 127-131. This sentence is very complex and not well written.**

[Response]

I reduced the complexity of the sentence. Please confirm. (Lines 127-132)

**Lines 164-167. I am sorry but I do not understand these sentence…**

[Response]

I reduced the complexity of the sentence. Please confirm. (Lines 168-171)

**Line 165. Change shingle to single.**

[Response]

Yes, thank you for pointing it out. It was a simple spelling mistake. We have revised. (Lines 170)

**With the term CMJ most of the researchers refer to a jump with the hands placed on the waist. In the present study, the authors used a CMJ. This should be made clear even from the introduction and I suggest the use of CMJas as an abbreviation in order to be easier for the reader to understand exactly the type of the jump used.**

**Why did the subjects executed only two trials of CMJs? Are the authors confident that they recorded accurately the vertical jump ability of the subjects. Line 199. Why the authors analyzed only the best jump and not the average of the two jumps. Wouldn’t this be more representative of subject’s ability?**

[Response]

I had changed CMJ to CMJAS, RJ to RJAS and, VJ to VJAS. we have described a protocol for the number of VJAS trials (Line 175). To measure many athletes within a limited schedule, we seated at least two successful VJAS trials. In addition, we conducted at least three familiarization trials while monitoring performance variables to ensure reliability and validity. We have revised the manuscript (Lines 172-173, Lines 175-180). Using the average of two trials may underestimate the participants' abilities, so we considered it necessary to analyze the "best jump" to evaluate the highest level of performances that participants could achieve at the test

**Lines 278-281, 289, 294-295…. I am confused… At the methods section the authors write that “The IRFDs were obtained as rates of increases in force across 50 ms in the interval from the onset to 250 ms as shown in Fig 3 (IRFD0–50, IRFD50–100, IRFD100–150, IRFD150–200 and IRFD200–250)”. The same terminology is used at the tables. When I read IRFD100-150 I understand that the change in force from 100 ms to 150 ms is calculated. However, the authors in the discussion write for example, IRFDs 0-150ms. When I read this I understand that the change in force from the onset to first 150 ms is calculated. This is different from 100-150ms. What is correct? The authors should make this clear from the methods section and consistent in the abbreviations they use throughout the text.**

[Response]

In the discussion, the term "IRFDs 0-150ms" refers to the IRFDs calculated for three separate time intervals: IRFD_0-50_, IRFD_50-100_, and IRFD_100-150_. As pointed out, We have standardized the wording throughout the entire text to either “IRFD_0-50_, IRFD_50-100_, and IRFD_100-150_” or expressions that correctly identify the IRFDss used in this study, such as “IRFDs were calculated from onset to ~ ms”, “IRFDs during onset to ~ ms ”, “IRFDs durig onset to ~ ms ” and so on (Lines 307-310, Lines 334-335, Line 348, Line 393, Line 409, Line 411, Lines 427-431).

**In the discussion, the authors based on for the significant correlations they found between the measured IRFDs and vertical jump variables try to explain the reasons for these relationships. Though, significant correlations can be found when a large sample of subjects is measured as the authors did in the present study and they are commended for that. A significant correlation does not mean a large effect as well. In the present study, only small to moderate correlations were found (0.165 – 0.457) between the iRFDs measured at different time intervals and VJ performance variables. Therefore, IRFDs explain from only 2.7 up to 20.9% of the variance in VJ performance parameters. When the partial correlations are considered, the significant correlations range from 0.174 to 0.362 explaining only 3 to 13% of the variance in VJ performance. Therefore, I do not agree with the approach the authors follow in the discussion where the importance these type of testing is overemphasized while the results do not support that. Based on my previous comment I do not agree with lines 372-379 and 385-387.**

[Response]

We have revised the manuscript based on your review. Please confirm (Lines 414-418, Lines 424-426, Lines 427-437).

**Table 1. Change “Index” to “Reactive strength index”**

[Response]

I had changed “Index” to “Reactive strength index” or ”RSI”. Please confirm. (Table 1-5, Lines 210…)
