# Supplementary material for: Association of multi-phase rates of force development during an isometric leg press with vertical jump performances
Source: PLoS One. 2024 Feb 21;19(2):e0292428. doi: 10.1371/journal.pone.0292428 (PMC10880963; doi:10.1371/journal.pone.0292428)
Supplement: Attachment — Submitted filename: Response to Reviwer.docx [file pone.0292428.s004.docx]

**Response to the comments**

Manuscript ID# PONE-D-21-32499

The title of manuscript:

Association of multi-phase rates of force development during an isometric leg press with vertical jump performances

Reviewer #1: I think the authors for the completed revisions, I have several further comments and suggestions.

----------------------------------------------------------------------------------------------------------

**Response to Reviewer # 1**

----------------------------------------------------------------------------------------------------------

**Ln202-204; Can the authors please include a justification for why only the dominant leg GRF was used for comparison to the ISLP data which is described as using bipedal force production? Quite simply the strength of the relationships could have been influenced by this change in seeking to compare the dynamic force production capabilities of a single leg to the force generation capacity of both legs in a different position/set up.**

[Response]

Taking into consideration the characteristics of single-leg and double-leg movements and intending to make participants’ measurements easier (line 134-136 and line 168-173). we employed the single leg press and bilateral VJ in this study. In the date processing for VJs, we used the dominant leg (line 207-208) that was used for isometric leg press measurement (line 136-137).

**Ln213; Please confirm that the data was checked for normal distribution prior to conducting the Pearson's correlation analysis.'**

[Response]

Several variables did not exhibit a normal distribution upon analyzing the normality (p > 0.05, line 224-225). Various sports athletes participated in the study can attributed to the results. Therefore, we have reanalyzed suitable statistical analyses (line 224-230). It is worth noting that the reanalysis did not yield significant changes in the results (line 233-290, Table1-5).

**Ln309-311; While I agree with the statement from the authors, it needs to be clarified that the VJ data was from a single dominant leg compared to bipedal force production and as such has likely influenced the strength of the correlation coefficients.**

[Response]

If we can improve the homogeneity of the participants and make the positions and/or setups of VJ and ISLP more similar, there is a possibility that the relationship between VJ and rapid force production capabilities could become stronger. I have added the topic (line414-416).

Reviewer #2: I thank the authors for considering my suggestions. The manuscript has improved significantly, and now the discussion section is better based on the findings of the study

----------------------------------------------------------------------------------------------------------

**Response to Reviewer #2**

----------------------------------------------------------------------------------------------------------

**Still, I believe that the whole manuscript should be checked very carefully for grammar and syntax issues and that there are several parts that are unclear to the reader (for example, lines 204-206, lines 220-222, lines 235-238, lines 315-318, lines 426-428).**

[Response]

We had the manuscript proofread by an English editing service and have checked the sentences clearer in terms of grammar and syntax.

**Lines 222-224: what is the purpose of these sentence in the statistical analyses section?**

[Response]

These sentences are the responses to comment form the other reviewer, and their purpose is to justify the reliability and validity of VJ measurements (line 219-221).

**Line 228: I cannot understand what this is.... In the methods it is written that a standard force value of 100N was used as a pretension. If it was a standard value for all the participants how a correlation would exist? Maybe I do not understand something, and I would like the authors to explain better this issue.**

[Response]

In this study, we instructed the participants to perform pre-tension approximately 100 N through visual estimation using the monitor (line149-151). This does not imply a requirement for the precise execution of a 100 N pre-tension. Because precise adjustments of muscle strength could potentially lead to neural inhibition and have a negative impact on explosive force generation. Therefore, considering the comment from the other reviewer, we investigated the influence of pre-tension that was not precisely 100N on the measurement values (line 238-240, Table 2, line 312-326).

**Table 1. First line, it should be 95% CI and not 95% IC.**

[Response]

Tank you for pointing it out. It was a simple spelling mistake. I corrected it (Table 1).
