# Supplementary material for: Association of multi-phase rates of force development during an isometric leg press with vertical jump performances
Source: PLoS One. 2024 Feb 21;19(2):e0292428. doi: 10.1371/journal.pone.0292428 (PMC10880963; doi:10.1371/journal.pone.0292428)
Supplement: Attachment — Submitted filename: Response to Reviwer.docx [file pone.0292428.s005.docx]

**Response to the comments**

Manuscript ID# PONE-D-21-32499

The title of manuscript:

Association of multi-phase rates of force development during an isometric leg press with vertical jump performances

The review should focus on two points:

**1. Answering the point made by Reviewer 1 with regards to the issues and potential limitations of comparing bilateral to unilateral tasks. Although an addition was made (Lines 414-416), the addition is not satisfactory as it does not really address this important issue. Please revisit this with a rationale for this selection (in Methods) and add any limitations this could cause (in the Limitations).**

[Response]

Thank you for pointing it out. In methods, I have reconsidered the reasons for using the double-leg vertical jump (VJ) and the single-leg leg press (line 134-139, 172-177). In addition to scientific rationale, these measurement designs were chosen because of considering how to enable diverse sports of participants to easily attain high performance in this measurement. Furthermore, I have suggested through the reference of a previous study that there are no critical issues of comparing bilateral to unilateral tasks(line 178-180).

However, there are some issues, I have added the limitations, including the comparison between bilateral and unilateral tasks (line 430-437).

**2. I appreciate the revised manuscript benefited from proof-reading. There remain, however, several passages where language is still difficult to follow, which might result in the point you are conveying being unclear. I would therefore, suggest that the revision includes another proof-reading, to ensure clarity of language for all points made.**

[Response]

We had the manuscript proofread by an English editing service and have checked the sentences clearer in terms of grammar and syntax.
